# Curcumin as theranostics: imaging-guided therapeutics in oncology and neurodegenerative diseases

**DOI:** 10.3389/fnut.2026.1790555

**Published:** 2026-06-12

**Authors:** Mohammad Yasir, Rahul K. Maurya, Alok S. Tripathi

**Affiliations:** 1 School of Pharmacy, ITM University, Gwalior, Madhya Pradesh, India; 2 Amity Institute of Pharmacy, Amity University Uttar Pradesh Lucknow Campus, Lucknow, India; 3 Department of Pharmacology, Era College of Pharmacy, Era University, Lucknow, Uttar Pradesh, India

**Keywords:** Alzheimer’s disease (AD), curcumin, neurodegenerative diseases, oncology, theranostics

## Abstract

Curcumin, a naturally occurring polyphenolic compound isolated from *Curcuma longa*, has been extensively studied for its nutritional, antioxidant, anti-inflammatory, anticancer, and neuroprotective properties. While traditionally recognized as a dietary bioactive with therapeutic relevance, recent advances in nanotechnology, molecular imaging, and targeted drug delivery have positioned curcumin as a promising theranostics agent. Theranostics integrates diagnostic imaging and therapy within a single platform, enabling real-time visualization of disease processes alongside targeted intervention. This review critically examines the emerging role of curcumin in imaging-guided therapeutics, with particular emphasis on oncology and neurodegenerative disorders. Curcumin’s intrinsic fluorescence, affinity for pathological proteins, and modulatory effects on key molecular pathways underpin its potential as both an imaging probe and a therapeutic molecule. Key challenges related to poor aqueous solubility, rapid metabolism, and limited bioavailability are discussed, along with innovative delivery strategies such as nanoparticles, liposomes, radiolabelled conjugates, and stimulus-responsive systems designed to enhance clinical applicability. By bridging its established nutritional significance with advanced theranostics applications, curcumin exemplifies the translational potential of plant-derived bioactive in precision medicine. This review highlights current progress, limitations, and future perspectives, positioning curcumin as a model phytoconstituent for the development of integrated diagnostic and therapeutic platforms in complex chronic diseases.

## 1 Introduction

Theranostics refers to the combination of diagnostics and therapeutic agents into a single chemical entity (1). The two components of theranostics are the diagnostic moiety, which provides an imaging signal following administration and indicates the location of the therapeutic moiety, and the therapeutic agent, which has a pharmacological effect that is location dependent (2, 3). The concept is highly relevant to both oncology and neurodegenerative diseases because these contexts lend themselves readily to imaging and therapy using the same compound (4–6). In addition to the matching of pharmacokinetics, the evolution of the concept has defined specific criteria for the evaluation of theranostic pairs, namely simultaneous determination of imaging and therapeutic responses, independent evidence for the relationship between imaging and therapeutic effects, mechanistic understanding of action, and ability to derive additional information from the imaging approach (7–9).

The theranostic concept continues to attract attention but remains underexplored in clinical translation because some imaging modalities and therapeutic pathways do not yet fully link imaging readout to therapeutic response and vice versa and therefore lack those additional degrees of freedom (8, 10). Nevertheless, concerns relating to safety and regulatory affairs particularly relevant for curcumin analogs have motivated an additional look at the topic. Establishing a pharmacological profile exposing the connection between an imaging action and the anticipated therapeutic activity can provide fresh insights on compound selection and formulation (11–13, 235).

## 2 Conceptual framework of theranostics

Theranostics a portmanteau of therapeutic and diagnostic refers to multimodal agents that integrate diagnosis, assessment of treatment response, and therapy into a single compound or formulation (10, 14). With theranostic agents, imaging and treatment decisions are guided by the same physiological target, and feedback from diagnostic readouts can influence both the choice of therapy and the dosage applied (10, 15, 16). The concept supports a variety of different imaging modalities (e.g., optical, nuclear, magnetic resonance) and therapeutic mechanisms (e.g., small molecules, antibodies, genes, chemotherapeutics) (17, 18). Research indicates that curcumin exhibits promising two-photon fluorescence properties, with two-photon absorption action cross-sections of 6 GM (tetrahydrofuran) and 2 GM (dimethyl sulfoxide) levels similar to rhodamine 6G. Evidence from confocal microscopy further supports its utility, showing efficient cellular internalization and establishing curcumin as a viable, biocompatible marker for two-photon imaging (19). Studies on curcumin-loaded solid lipid nanoparticles (C-SLNs) have confirmed their ability to target the brain following significant improvements in pharmacokinetic profiles. Through fluorescence imaging and biodistribution studies using {99m}Tc-labeled compounds, C-SLNs were shown to effectively traverse the blood–brain barrier. The nanoparticle formulation exhibited a thirty-fold higher brain AUC ratio compared to free curcumin, alongside an eight-fold increase in oral bioavailability. These data support the potential of C-SLNs as a theranostic platform, serving both as a non-invasive SPECT imaging agent for solid tumors and a carrier for direct brain delivery of curcumin (20). Further integration of magnetic nanoparticles with natural fluorophores like curcumin offers a promising route for multifunctional theranostics. In one instance, a miniemulsion polymerization technique was used to embed superparamagnetic iron oxide nanoparticles and curcumin into a polymer shell, resulting in nanoparticles that exhibited both superparamagnetism and bright fluorescence. With intrinsic loss power values suitable for magnetic hyperthermia and proven non-toxicity in osteosarcoma cell lines, these systems effectively demonstrate the potential of hybrid nanomaterials. They are proposed as viable tools for cell tracking and multimodal treatment strategies involving MRI and photodynamic therapy (21). These characteristics make curcumin a versatile candidate when considered for theranostic use in oncological or neurological applications (22–24).

The potential value of targeted and imaging-guided therapies has been recognized for some time. However, remaining compliance issues, such as assurance of safety and material quality, need to be taken into account (22, 23). Theranostic agents must, for example, fulfill regulatory requirements and need clear evidence of effectiveness in patients to encourage adoption. The assessment of cancer therapy is still in early stages. Currently, improvement in patient outcome is generally accepted as the gold-standard parameter of therapeutic or imaging agent effectiveness (as a result, confirmation through controlled clinical trials is also anticipated) (24, 25).

Curcumin, a natural polyphenolic molecule with anti-inflammatory, antioxidant, and anticancer effects, has been thoroughly investigated in theranostic applications. These systems integrate therapeutic effects (e.g., inducing apoptosis, modulating inflammation, or functioning as a photosensitizer) with diagnostic imaging capabilities, frequently utilizing nanoformulations such as lipid nanoparticles, polymeric micelles, iron oxide nanoparticles, or dendrimers to enhance bioavailability and targeting (26). Researchers have incorporated curcumin into systems utilizing numerous imaging modalities, occasionally in multimodal setups.

### 2.1 Optical imaging (fluorescence, near-infrared/NIR)

The intrinsic fluorescence of curcumin, frequently augmented or altered in derivatives or nanoparticles, renders it appropriate for optical monitoring of medication administration, cellular absorption, or disease biomarkers such as amyloid-β (Aβ) plaques. Optical modalities provide excellent sensitivity and real-time functionality, although they are constrained by tissue penetration depth. NIR fluorescent curcumin analogues, such as donor-acceptor-donor structures or CRANAD-related chemicals, facilitate *in vivo* imaging of Aβ species in Alzheimer’s disease animal models via the intact skull, exhibiting “off–on” fluorescence for enhanced target-to-background ratios. These function as multitarget theranostic drugs, integrating imaging capabilities with possible neuroprotective properties (27). Curcumin-encapsulated upconversion nanoparticles (UCNPs) within PLGA matrices for cancer theranostics provide near-infrared imaging in conjunction with photodynamic or therapeutic effects (28). Curcumin and its conjugated systems function as fluorescent probes for cellular tracking or monitoring release in nanoparticles, utilizing its robust inherent fluorescence (29). In the field of nanotheranostics, fluorescence imaging is combined with the biosensing of tumor biomarkers (e.g., HER2, EGFR) or photoacoustic imaging (26).

### 2.2 Nuclear imaging

Nuclear approaches offer great sensitivity and quantitative monitoring of biodistribution, frequently employing radiolabeled curcumin-loaded carriers (30–32). Nuclear imaging is proficient in comprehensive quantitative assessment and the possibility for theranostic pairing (e.g., transitioning from diagnostic to therapeutic radionuclides), despite the use of ionizing radiation. Curcumin analogues such as DiFboron-8 (or [^18^F]-DiFboron-8) serve as dual fluorescent/PET probes with a strong affinity for Aβ aggregates in Alzheimer’s disease animal models (APP/PS1), facilitating early identification (33). ^99m^Tc-radiolabeled hyaluronic acid/TPGS-based curcumin-loaded nanoparticles for breast cancer theranostics facilitate nuclear imaging in conjunction with synergistic therapy (34). Multifunctional lipid nanoparticles encapsulating curcumin utilized for the stabilization of atherosclerotic plaques utilize single photon emission computed tomography (SPECT) in conjunction with MRI to assess accumulation and macrophage polarization (35, 36). Radiolabeled polymeric nanoconstructs co-loaded with docetaxel and curcumin facilitate PET imaging for combinatorial cancer therapy and efficacy evaluation (37).

### 2.3 MRI (magnetic resonance imaging)

MRI is non-ionizing and provides great resolution, although exhibits poorer sensitivity compared to nuclear or optical techniques. MRI offers superior soft-tissue contrast and anatomical detail, often augmented by superparamagnetic iron oxide nanoparticles (SPIONs/USPIOs) or gadolinium complexes co-formulated with curcumin (38). Curcumin-conjugated superparamagnetic iron oxide (SPIO) nanoparticles (e.g., SDP@Cur-CRT/QSH with peptides for blood–brain barrier penetration and amyloid-beta targeting) facilitate T_2_-weighted MRI visualization of amyloid plaques in Alzheimer’s disease while offering NLRP3 suppression for cognitive enhancement (39). Curcumin-Fe_3_O_4_@ZIF-8 nanoformulations serve as pH-responsive T_2_ MRI contrast agents, exhibiting pH-dependent improvement in r_2_ relaxivity values for theranostics in triple-negative breast cancer, integrating imaging, reactive oxygen species production, and radiosensitization (39). Curcumin-coated ultra-small superparamagnetic iron oxide (USPIO) nanoparticles function as T_1_ contrast agents for MRI of cancer cells, utilizing curcumin’s therapeutic attributes (40). Multifunctional magnetic nanoparticles (e.g., Fe_3_O_4_@PLA–PEG or MIL-Cur systems) provide the delivery of curcumin with remote magnetic field-regulated release while improving MRI contrast (41). Apoferritin infused with curcumin and gadolinium-based contrast agents (e.g., GdHPDO3A) for “theranostic” MRI in certain applications (42).

## 3 Pharmacological profile of curcumin

Curcumin exhibits suboptimal pharmacokinetics for clinical translation in imaging-guided theranostics. The compound’s poor bioavailability, rapid metabolic degradation, and extensive plasma protein binding severely limit the diagnostic and therapeutic applications of curcumin-based probes and formulations (43). Liquid chromatography–tandem mass spectrometry (LC–MS/MS) analysis of blood plasma from healthy human subjects reveals an elimination half-life of only 1.84 h following a single intravenous administration of 200 mg curcumin; no curcumin was detectable beyond 24 h (12, 44). Consequently, both contrast agents and therapeutic compounds fail to accumulate in target tissues at therapeutically effective concentrations, resulting in minimal imaging signals and limited pharmacological activity.

Formulation strategies to enhance the bioavailability of curcumin include the use of solid dispersions, liposomes, polymeric nanoparticles, and other nanocarriers. Formulated curcumin acquires high target-tissue delivery, significantly overcomes biological barriers, and enhances imaging signals in both preclinical and clinical settings (26–28). Nanoparticle-, micelle-, liposome-, and conjugate-based formulations of curcumin constitute well-established, maintained vectors for the targeted diagnostics of oncological and neurodegenerative diseases (231). Fully characterized at the pharmacokinetic and formulation levels, other surface-engineered therapeutic agents and nanocarriers may integrate curcumin into theranostics still in strong demand (26, 29, 30). Equally important are the established therapeutic targets and traceable cellular alterations, to which effective curcumin concentrations deliver diagnostic readouts.

The therapeutic activity of curcumin stems from direct and indirect modulation of several prominent molecular targets associated with oncological and neurodegenerative diseases (31, 32, 43, 234). Indirect means include the up-regulation and down-regulation of activator and inhibitory proteins, respectively, that control the levels of the well-known targets (33, 34).

Surface engineering of nanocarriers has become essential to overcome the pharmacokinetic limitations of curcumin and to realize its full potential in theranostics. By incorporating polyethylene glycol (PEG) coatings, or “stealth” layers, researchers have successfully prolonged the circulation half-life of curcumin formulations. For example, Song et al. utilized PLGA-PEG-PLGA triblock copolymeric micelles to encapsulate curcumin, reporting significantly improved pharmacokinetics and enhanced *in vivo* distribution facilitated by the Enhanced Permeability and Retention (EPR) effect (45). To further increase specificity, active targeting strategies have been employed to direct nanocarriers to cancer cells via receptor-mediated endocytosis. Notably, the development and evaluation of folic acid-conjugated curcumin-loaded functionalized multi-walled carbon nanotubes demonstrated enhanced efficacy in ovarian cancer treatment, confirming that such surface modifications significantly improve targeting capabilities (46).

Furthermore, the development of PLGA-lecithin-PEG nanoparticles modified with RNA aptamers demonstrates their ability to selectively target epithelial cell adhesion molecule (EpCAM) receptors that are overexpressed in colorectal cancer, consequently enhancing cellular uptake and therapeutic effectiveness compared to non-targeted formulations (47). Curcumin-loaded lipid-polymer-lecithin hybrid nanoparticles were synthesized and functionalized with ribonucleic acid (RNA) Aptamers. These curcumin-encapsulated bioconjugates were characterized for particle size, zeta potential, drug encapsulation, stability, and release. Gold nanoparticles (AuNPs) have been produced utilizing curcumin as both a reducing and capping agent, establishing an environmentally friendly theranostic platform. These particles utilize surface plasmon resonance (SPR) for imaging and photothermal therapy while administering the therapeutic payload (48, 233). Furthermore, upconversion nanoparticles (UCNPs) encapsulated in PLGA have been infused with curcumin; these nanoparticles convert near-infrared light (980 nm) into visible light to activate curcumin’s photodynamic toxicity deep within tissues, thereby addressing its restricted penetration depth (28, 49).

The capacity to modulate these targets provides curcumin with an extensive spectrum of anticancer properties that are easily traceable at the cellular level. Limited combinations with other treatment modalities maximize the theranostic potential of unformulated curcumin and extend the applicability of highly characterized derivatives and surfaces (35–37).

### 3.1 Bioavailability and formulation advances

Theranostics, a portmanteau of therapy and diagnostics, is a drug development paradigm that aims to integrate diagnostics and therapeutics in a single agent. The pertinent definition is that the agent possess both diagnostic and therapeutic functions, referred to as the theranostic pair (38). Although theranostics was first applied in radioactive agents, the concept has become increasingly relevant with the rise of molecular imaging and targeted therapy in cancer (38–41, 237). It draws attention not only to the diagnosis of diseases but also to the prediction of treatment success and the monitoring of therapeutic efficacy.

The strong interest in theranostics is driven by the clinical need to better match patients with appropriate therapies, thus optimizing an increasing number of treatment choices available on the market. The concept is also applicable for other diseases, such as neurodegenerative conditions like AD where either imaging or biomarker-based diagnostics can guide treatment selection (42, 44, 50). Nevertheless, the stringent regulatory framework surrounding the development of combination drugs presents a significant barrier to the clinical translation of theranostics. Data supporting device Guidance for Industry provides detailed consideration when pursuing such an optimal but challenging plan (12, 51).

Curcumin belongs to the dietary polyphenol family and is a natural pigment widely present in *Curcuma longa*, or turmeric. It serves as a pharmacologically active component possessing antioxidant, anti-inflammatory, and anticancer properties with a long history of safe consumption (52, 53, 232). Curcumin does so at remarkably low concentrations (ng to μg per mL in the systemic circulation). Its predominant pharmacological effects include reduction of cell proliferation, induction of apoptosis, suppression of angiogenesis and invasion, modulation of cancer stem cells (CSCs), and inhibition of neuroinflammation (54, 55). It exerts beneficial effects along multiple pathways in cancer and AD development. Therefore, curcumin has emerged as one of the most promising theranostic agents (56, 57).

Curcumin has been widely studied for its anticancer potential in various malignancies, including breast, colorectal, lung, and prostate cancers, owing to its ability to modulate multiple signaling pathways involved in tumor progression (242). In combination with the anticancer agent paclitaxel, curcumin has been considered a promising candidate for theranostic imaging and therapy in breast cancer (58). Curcumin is also actively investigated for its role in the therapy of AD, including combination with anti-inflammatory agents (59, 60). AD therapeutics face major challenges due to limited success in late-phase clinical trials. Curcumin-containing formulations have entered clinical trials for AD, and exploration of curcumin as a component of AD theranostics has started (61, 62).

Nonetheless, curcumin has poor bioavailability due to its rapid metabolism and elimination, severely limiting its clinical use. To overcome such challenges, advances in delivery systems have been explored extensively and the following solid dispersion systems, polymer-based micro/nanoparticle formulations and liposomal delivery vehicles have shown promise in enhancing curcumin solubility and bioavailability (24, 63, 64). The incorporation of curcumin into solid dispersions has been found to provide a three- to fifteen-fold improvement in aqueous solubility and upto 230-fold enhancement of bioavailability (24, 65).

### 3.2 Pharmacodynamics and molecular targets

Curcumin interacts with various molecular targets associated with both diagnostic imaging and therapeutic responses, including NF-κB, signal transducer and activator of transcription 3 (STAT3), cyclooxygenase-2 (COX-2), the amyloid precursor protein and Aβ peptide (APP/Aβ), and amyloid and tau pathways (66–68). Although many therapeutic targets modulated by curcumin support its use in imaging-guided therapy, it also harbours additional targets relevant only to diagnostic imaging or therapy alone. Together, these interactions underscore curcumin’s theranostic potential (69).

Curcumin interferes with the NF-κB signalling pathway and inhibits STAT3 activity, both of which reduce the transcription of pro-inflammatory cytokines and are associated with positive outcomes in cancer and neurodegenerative diseases (70). The compound also suppresses COX-2 expression, which is generally upregulated in cancerous tissue and promotes unabated proliferation of cancer cells, metastasis, resistance to apoptosis, and angiogenesis (71). APP overexpression and the consequent production of toxic Aβ peptides are characteristic of the Alzheimer’s disease (72). In Alzheimer’s disease, “amyloid-β (Aβ) levels” refer to the concentration and distribution of Aβ peptides, mainly Aβ_40_ and the more aggregation-prone Aβ_42_, across biological compartments (73). These include soluble monomers and oligomers as well as insoluble fibrillar aggregates deposited as plaques (74). Soluble oligomeric Aβ species are considered the most neurotoxic, whereas fibrillar forms contribute to plaque burden (75, 76). Aβ levels are commonly quantified using biochemical assays such as ELISA for soluble fractions (77, 78), and immunohistochemistry for tissue deposition. Thus, Aβ levels represent a composite measure of peptide concentration, aggregation state, and spatial distribution relevant to disease progression.

**Figure 1:**
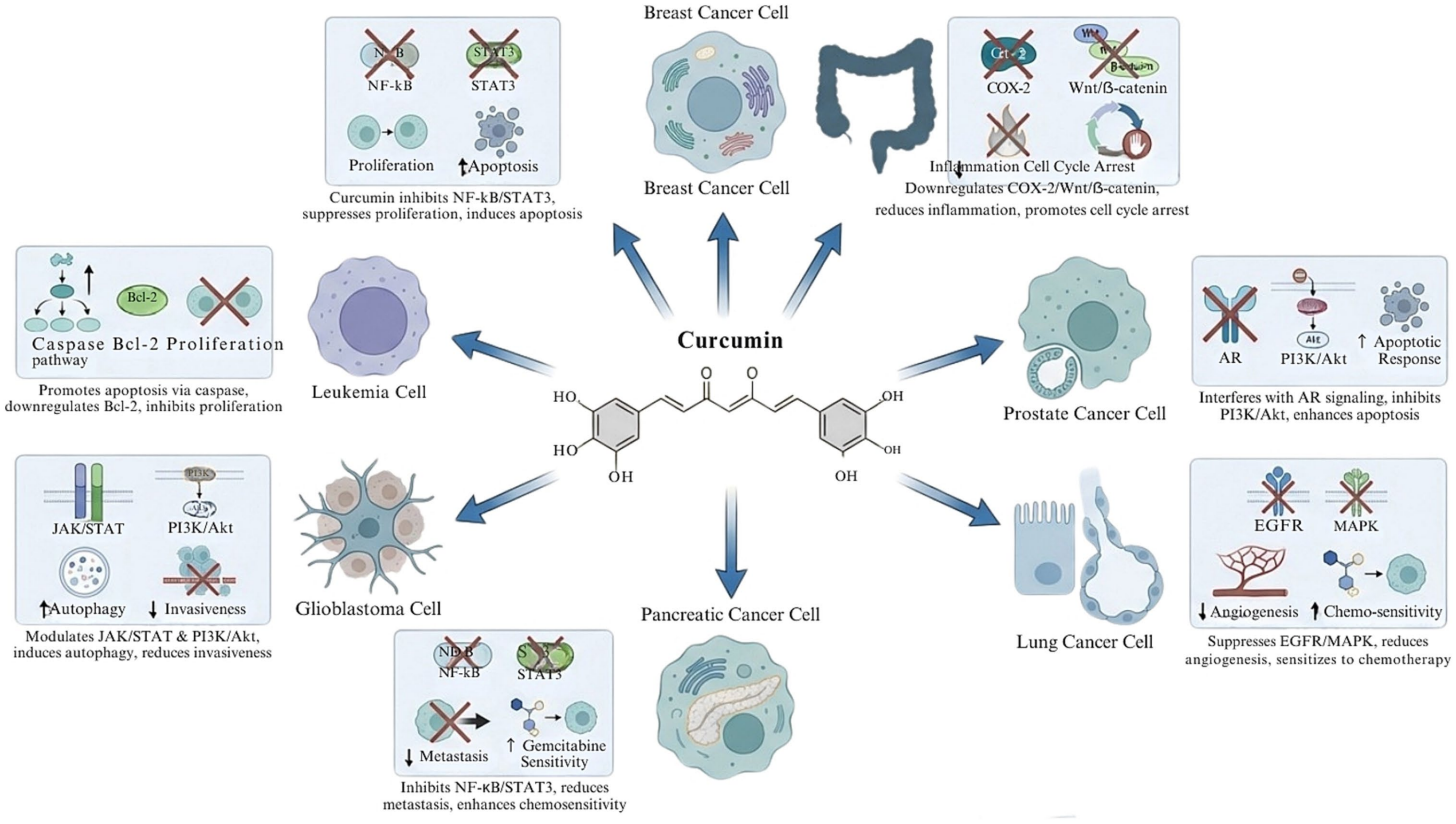
Anticancer mechanisms of curcumin across multiple malignancies.

Curcumin reduces both APP and Aβ levels and aids the modelling of various cellular and molecular events within AD, including the amyloid and tau pathways. Curcumin upregulates the apoptotic BMI-1 protein and downregulates various anti-apoptotic proteins; enhances mitophagic and autophagic clearance of damaged and aggregated proteins; and modulates neuroinflammation and redox homeostasis (79) (Figure 1).

The specific molecular pathways and modes of action through which curcumin interacts with, regulates, or prevents amyloid aggregation involve a complex interplay of signaling cascades (80). Central to this mechanism is the modulation of the PI3K/Akt/mTOR axis, where curcumin activates Akt to inactivate GSK-3β, a critical kinase responsible for tau hyperphosphorylation and neuronal damage; this inactivation subsequently reduces Aβ production and plaque formation while positively influencing cholinergic function (81). Furthermore, curcumin exerts neuroprotective effects by inhibiting the ROS/JNK pathway, thereby mitigating oxidative stress and neuronal apoptosis, and by suppressing phospho-mTOR to lower Aβ levels (82). The compound also directly targets Aβ generation by downregulating Beta-secretase 1 (BACE1) expression (83, 84). Regarding neuroinflammation and immunity, curcumin inhibits the activation of NF-κB to reduce inflammatory responses and modulates the TREM2/TyroBP pathway involved in innate immune activation. Additionally, curcumin influences transcription factors such as GABPa, PPARγ, TFAM, and PGC-1, which are pivotal for maintaining mitochondrial function in Alzheimer’s disease models.

Curcumin interacts with amyloid species in the grooves of structured fibrils and with disordered or monomeric Aβ, generating fluorescence upon accumulation at deposits while concurrently inhibiting β sheet formation and aggregation (62, 63). The precise binding stoichiometry remains undefined, and the universality of a singular binding mechanism is uncertain. Curcumin interacts with amyloid aggregates via specific, morphology-dependent interactions, illustrating how the same molecule can associate with both plaques and smaller Aβ species (64). Molecular dynamics and biochemical studies demonstrate distinct groove binding on ordered fibrils and a more disordered, composite arrangement with monomers or nonfibrillar aggregates (24, 65).

Curcumin exhibits intrinsic fluorescence, with its optical signal significantly influenced by the surrounding environment and its interaction with Aβ species, a property utilized in imaging probes based on curcumin (66). Curcumin-based probes exhibit significant alterations in fluorescence properties when combined with both soluble and insoluble Aβ, with signal amplification occurring at deposit sites. The fluorescence intensity and spectrum characteristics alter when curcumin or curcumin-derived probes interact with soluble and insoluble Aβ species, signifying binding-dependent optical activation (65, 67). Curcumin’s self-association and affinity for Aβ can concentrate fluorescent molecules at plaques, hence improving detectability (65).

The identical contacts that localize curcumin to aggregates often yield the necessary functional results for inhibition, resulting in imaging and anti-aggregation activities that commonly originate from overlapping binding mechanisms (68). Experimental and modeling studies indicate that curcumin binding both identifies plaques and inhibits or reverses aggregation events (63). Curcumin inhibits the development of oligomers and fibrils and can disassemble existing aggregates, aligning with its binding that disrupts backbone hydrogen bonding and β sheet organization. Deposition within fibril grooves can sterically and electrostatically modify the fibril surface, potentially obstructing monomer incorporation or additional lateral assembly (24, 69). Curcumin scaffolds can be altered to position functional groups adjacent to critical residues, such as histidines, to prevent metal-induced cross-linking and covalent stabilization of aggregates, illustrating that scaffold binding can be redirected to obstruct specific aggregation routes (70–72).

Collectively, binding events that provide a fluorescence signal frequently align with the molecular interactions that restrict β sheet formation and fibril growth; hence, in numerous instances, the imaging-capable binding also facilitates inhibition (73). Although curcumin’s dual roles in imaging and inhibition are substantiated, significant molecular details remain ambiguous in the existing literature and may differ according on scenario. Variations in aggregate form, local concentration, membrane environment, and metal ions affect both signaling and inhibitory efficacy, and not every binding event is assured to exhibit high fluorescence and impede aggregation. Curcumin can influence peptide aggregation routes through membrane partitioning, offering an indirect inhibitory mechanism that differs from direct peptide binding (74). The equilibrium between curcumin self-aggregation and peptide affinity influences local probe concentration, hence affecting both optical readout and inhibitory effectiveness (65). Evidence indicates overlapping binding mechanisms that allow curcumin to serve concurrently as an imaging probe and an aggregation inhibitor; nonetheless, quantitative stoichiometry and applicability across all amyloid situations remain unaddressed in the existing literature (65, 75).

## 4 Curcumin in diagnostic imaging

Disclosure of physiological changes and non-invasive assessment of treatment effects are key objectives of therapy. Theranostics fulfills these objectives by cross-talking between imaging and therapy treatment efficacy may be visualized through the same modality as used for detection, such as within the “pharmacodiagnostics” nomenclature.

Curcumin undergoes extensive hepatic metabolism after oral administration. Among the fundamental pharmacokinetic properties of curcumin is the low plasma concentration of curcumin itself, hence poor accessibility of target organs including brain and tumour alike (85). As a result, only the curcumin metabolites, such as DHC, may account for the imaging and therapeutic effects observed and, seeking the curcumin theranostic, elimination of curcumin from the formulation altogether becomes compelling. For instance, curcumin has been extensively studied for non-invasive imaging of Alzheimer’s-related brain pathologies, such as the amyloid-β debate whereas curcumin alters the accumulation of Aβ-42 or tau (86–89).

Despite its potential as an imaging probe, curcumin faces several limitations in neurodegenerative applications, including poor pharmacokinetics, rapid metabolism, and limited brain availability, which reduce signal reliability. Its moderate target specificity and multi-target interactions may also lead to non-specific binding. Furthermore, curcumin’s interaction with amyloid-β aggregation can interfere with accurate plaque visualization, while its suboptimal optical properties constrain robust *in vivo* imaging (90–92). Strategies such as structural modification and nanocarrier-based delivery are being explored to address these challenges.

### 4.1 Optical imaging approaches

Optical fluorescence and near-infrared (NIR) imaging are non-invasive techniques that guide surgical resection and ablation therapy, yielding improved clinical outcomes (93). Probes for cancer diagnosis and therapy can be developed from curcumin technology. Fluorescent organic dyes or quantum dots without additional conjugation retain their optical properties (94). Signal-to-noise ratio and target selectivity can be improved using conjugated probes; covalent modification of curcumin, including fluorinated derivatives, has been investigated to enhance imaging performance for amyloid detection, such as in ^19^F-MRI applications (95). Targeted and chemically modified curcumin-based probes have been explored to improve specificity and imaging performance in amyloid detection (62, 96, 97). Data demonstrate enhanced anticancer efficacy when curcumin is directly conjugated to therapeutic agents, allowing monitoring through fluorescence signals (98–100).

Radiolabelled curcumin analogues generally exhibit broad biodistribution, with predominant accumulation in the liver, spleen, and intestines and relatively low-to-moderate tumour uptake, along with rapid systemic clearance (30). Positron emission tomography (PET) allows spatiotemporal and quantitative analysis of the diagnostic and therapeutic roles of curcumin-based images in brain cancer models (101, 102).

Magnetic resonance imaging (MRI) enables deep tissue observation without ionizing radiation. T1 or T2 contrast agents including iron oxide, manganese, and gadolinium-based nanoparticles are typically used as enhancers (103–105). Curcumin or curcumin-derivative nanoparticles may enhance naloxone-loaded nanoplatform signals during therapeutic development for opioid overdose treatment (106). Control and curcumin-treated groups demonstrate distinct signal variations in animal imaging data, indicative of curcumin accumulation within nanocarriers. Talabardon nanoparticles loaded with curcumin stimulate anticancer action on human hepatocellular carcinoma and breast cancer models. Furthermore, integrated magneto-optical systems permit fluorescence and MRI combination for versatile targeting studies (107, 108).

### 4.2 Nuclear imaging and radiolabelled curcumin

Curcumin is a promising biomarker in AD research, and its fluorescent properties have allowed it to be used as a molecular probe for the detection of amyloid aggregates. Despite its inherent advantages, curcumin suffers from rapid clearance and extensive gut metabolism after administration (109, 110). These shortcomings compromise investigation of the compound’s contributions to AD diagnostics and therapy. Various formulation techniques such as the creation of micelles, nanoparticles, and liposomes have been developed to enhance the solubility and bioavailability of curcumin and boost the delivery of this therapeutic agent to specific tissues (111–113). Collectively, these strategies are expected to facilitate the detailed examination of curcumin’s complex interactions with amyloid and tau species, and to link these interactions with its broader role in combination therapy (114).

Isotopes within the curcumin family have been conjugated with radiolabels including ^18^F and ^99m^Tc at positions that preserve their activity, and advances in radiolabelling and formulation techniques have also led to the development of curcumin derivatives optimized for dual-function theranostics (30). These conjugates are anticipated to be particularly beneficial for studying the pharmacological mechanisms of curcumin at various sites in the body, as well as for monitoring the simultaneous impact of curcumin on multiple oncological and neurodegenerative pathways. The biodistribution of gallium-68-labeled DOTA-curcumin has been investigated with particular regard to colon-rectal carcinoma, and 18F-fluorocurcumin biodistribution has been evaluated through measurements of absorbed dose and effective dose for multiple cancer models (115–117, 236).

### 4.3 Magnetic resonance and other modalities

Curcumin galactoside exhibits favorable properties for investigational imaging, notably regarding nuclear modalities. Radiolabelling of curcumin and analogs enables PET imaging with isotopes such as 18F and single-photon computed tomography with ligands including ^99m^Tc, revealing pertinent information on biodistribution and dosimetry (26, 118, 119). Coupling gamma-emitting radionuclides to curcumin facilitates *in vitro* cellular tracer studies and large-animal models for theranostic evaluation (30).

The versatility of curcumin as a molecular probe across optical, nuclear, and magnetic resonance modalities enables simultaneous optical and nuclear imaging with corresponding precision (26, 120). Emerging magnetic resonance imaging (MRI) approaches exploit curcumin’s affinity for amyloid-β aggregates by incorporating it into nanoparticle-based contrast systems (e.g., gadolinium-conjugated systems), enabling targeted plaque detection and enhanced imaging contrast (40, 121, 122). In addition, curcumin-derived or curcumin-functionalized nanomaterials have demonstrated multimodal imaging capabilities, including combined fluorescence and MRI applications. While advanced spectroscopic techniques continue to be explored for biomolecular detection, the application of curcumin in such modalities remains at an early stage and requires further validation.

Emerging magnetic resonance schemes exploit curcumin’s multi-targeted pharmacodynamics to achieve imaging contrast. Terahertz techniques are reported. Despite substantial investigation of curcumin’s anticancer and anti-Alzheimer activity, scrutiny of parallel imaging capabilities remains limited (108, 123).

Curcumin demonstrates advantageous properties relative to numerous current imaging probes and therapeutic drugs for neurodegenerative diseases in preclinical models; however, it remains inadequate to approved drugs in terms of clinical specificity, sensitivity, and overall efficacy (124–126). Additionally, engineered curcumin-based fluorescent probes can identify Aβ and tau aggregates, along with reactive oxygen species *in vivo*, indicating strong target specificity and sensitivity for early-stage pathology. Multiple groups have developed curcumin-based NIR probes and analogues aimed specifically at Aβ detection rather than tau detection in these reports (127–129). Curcumin demonstrates extensive neuroprotective properties through anti-amyloid, anti-tau, antioxidant, anti-inflammatory, and metal-chelating mechanisms, showcasing significant efficacy in animal models of Alzheimer’s and other neurodegenerative disorders (110, 130). However, Curcumin decreased thioflavin T (ThT) signal and caspase activation in a three-dimensional human neuron/astrocyte spheroid model produced from induced pluripotent stem cells (iPSCs) of Alzheimer’s disease patients, signifying a reduction in Aβ aggregation and subsequent cell death signals (131). Nonetheless, its low oral bioavailability and limited success in clinical trials decrease its practical effectiveness compared to more refined, targeted pharmaceuticals, despite their favorable safety profile and diverse actions, which render it a promising foundation for next-generation imaging and therapeutic agents. Despite its potential as an imaging probe, curcumin faces several limitations in neurodegenerative applications, including poor pharmacokinetics, rapid metabolism, and limited brain availability, which reduce signal reliability (12, 132, 133). Its moderate target specificity and multi-target interactions may lead to non-specific binding compared with established tracers such as Pittsburgh Compound B and Florbetapir F 18 (134–136). Furthermore, curcumin’s interaction with amyloid-β aggregation, while therapeutically beneficial, may interfere with accurate plaque visualization and quantification (137, 138). In addition, its suboptimal optical properties and low signal intensity constrain robust *in vivo* imaging performance (9, 10, 96). Strategies such as structural modification (e.g., CRANAD analogues) and nanocarrier-based delivery are being explored to overcome these limitations and improve imaging reliability (24).

## 5 Curcumin as a therapeutic agent in theranostics

Curcumin serves as a versatile anticancer agent through multiple cellular and molecular signalling pathways, including modulation of inflammation, antioxidant activity, and direct interactions with key proteins. These effects have accompanied the development of strategies for theranostic applications that integrate curcumin-based imaging with therapeutic interventions (139, 140). Curcumin interacts with both Aβ and tau directly, targeting neuroinflammation, the Aβ pathway, and the tau pathway in a manner consistent with these neurodegenerative conditions and facilitating the establishment of non-therapeutic imaging-guided therapeutic frameworks (56, 141). Furthermore, the lack of dose-limiting toxicities in preclinical studies with curcumin has enabled the consideration of imaging and therapy integration. These attributes highlight curcumin’s capacity to combine imaging-guided and therapeutic strategies in both oncological and neurodegenerative disease contexts (142).

Molecular Aβ aggregation of amyloid precursor protein (APP) is initially cleaved by β-secretase (BACE1) within endosomes, producing the C99 fragment. C99 is then digested by *γ*-secretase, resulting in the release of Aβ peptides, particularly Aβ40 and Aβ42, into the extracellular area (143). Furthermore, released Aβ monomers self-assemble into soluble oligomers, which subsequently develop into fibrils and ultimately form amyloid plaques embedded in the extracellular matrix (144). It is important to clarify that soluble Aβ oligomers are the primary neurotoxic entities responsible for synaptic dysfunction, membrane disruption, and oxidative stress, particularly in the context of early aggregation (145). Physiological tau associates with microtubules and enhances axonal transport stability. Hyperphosphorylation transpires when kinases (e.g., GSK-3β, CDK5) exhibit heightened activity or when phosphatases are dysfunctional, resulting in excessive phosphorylation at particular serine/threonine residues (e.g., Ser396, Thr231) (146). Hyperphosphorylated tau dissociates from microtubules, misfolds, and self-assembles into paired helical filaments and neurofibrillary tangles (NFTs), which are highly associated with cognitive decline. Extracellular Aβ oligomers can activate intracellular kinases, such as GSK-3β, and inflammatory pathways that facilitate tau hyperphosphorylation (147). Aβ can initiate tau aggregation in cell-free and animal models, expediting NFT development and disseminating tau disease throughout brain areas (148). The “Aβ → tau→neurodegeneration” pathway supports a theranostic strategy aimed targeting both proteins (147). Curcumin may indirectly diminish APP processing or oxidative stress that facilitates amyloidogenic cleavage, hence influencing Aβ production (149). Curcumin can bind to Aβ and redirect its assembly from hazardous oligomers to less dangerous or non-toxic aggregates, while also safeguarding membranes from Aβ-induced permeabilization (149). Moreover, curcumin can impede Aβ-induced activation of the PTEN/Akt/GSK-3β pathway, thereby diminishing GSK-3β-mediated tau phosphorylation at critical locations including Thr231 and Ser396. Curcumin’s anti-inflammatory and antioxidant properties mitigate Aβ- and tau-induced neuroinflammation and oxidative damage, hence disrupting the Aβ-tau-neurodegeneration cycle (150).

### 5.1 Anticancer applications and mechanisms

**Figure 2:**
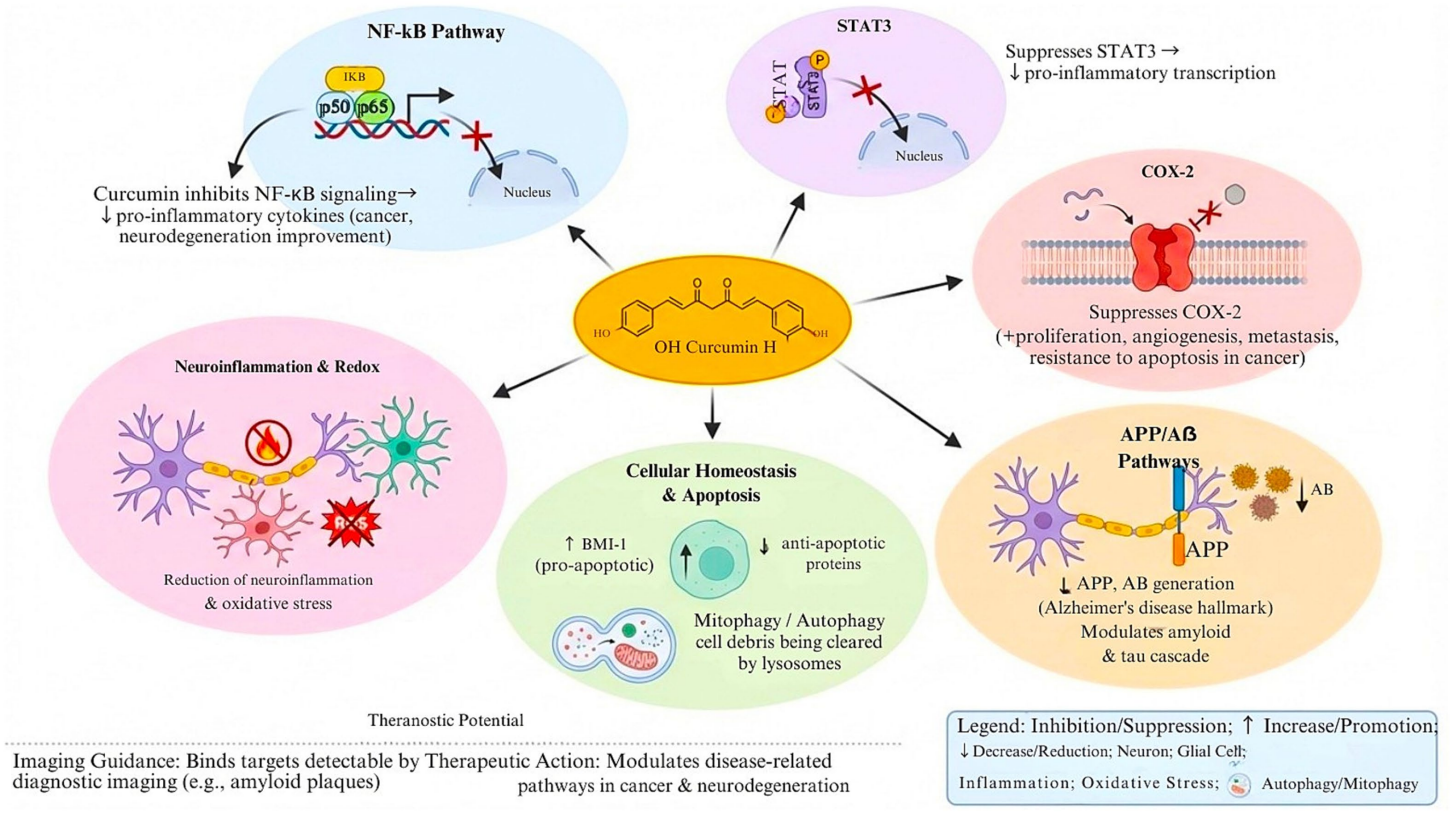
Multifaceted therapeutic potential of curcumin in cancer and neurodegenerative disorders.

Curcumin, a naturally occurring polyphenol extracted from the rhizome of *Curcuma longa*, has a long history of use in traditional medicine, particularly for cancer treatment (151). It exhibits a variety of biological properties, including anticancer, anti-inflammatory, antioxidative, and antimicrobial activities (152, 153, 241). The growing awareness of curcumin as a chemopreventive agent has also driven its application in imaging-guided therapy, as illustrated in Figure 2, which highlights its dual diagnostic and therapeutic roles. Advances in imaging techniques, such as optical, nuclear, and magnetic resonance modalities, are paving the way for curcumin to serve as an effective theranostic agent (154–156). To date, numerous studies have confirmed curcumin’s anticancer efficacy. In addition to suppressing cellular proliferation and inducing apoptosis, curcumin inhibits angiogenesis and the development of CSCs, exerting its anticancer effects through diverse cellular and molecular targets (Table 1). Overexpression of curcumin targets in malignant tissues also suggests the feasibility of developing curcumin-based imaging probes for monitoring therapeutic interventions (67, 157–159).

**Table 1:** Curcumin-based theranostics strategies in cancers.

S. no	Disease area	Target/mechanism	Curcumin analog/platform	Imaging modality/label	Clinical/preclinical relevance	Theranostic utility	References
1	Glioblastoma	HSP-70 inhibition, photothermal effect	NIR phototheranostic curcumin nanoplatform	NIR fluorescence imaging	Preclinical (GBM models)	Imaging-guided triple-modal therapy; survival extension	(220)
2	Oncology (glioma)	Radio-sensitization	Curcumin (non-radiolabelled, adjuvant platform)	Conventional imaging support (MRI/CT)	Preclinical & Clinical (adjuvant context) GBM models	Enhances radiotherapy efficacy; therapeutic sensitization rather than intrinsic imaging	(221)
3	Breast cancer	Tumour targeting via hyaluronan-based NP uptake (active targeting)	^99m^Tc-Radiolabelled HA-CHEMS-Cur-TPGS NP curcumin-loaded 6amphiphilic nanoparticle	SPECT/CT imaging with ^99m^Tc label	Preclinical (4 T1 tumour-bearing mice)	Combined tumour imaging and synergistic antitumour therapy with enhanced biodistribution and cytotoxicity	(34)
4	Breast cancer	Intravenous curcumin synergistically combined with chemotherapy/radiotherapy	Nano-emulsifying drug delivery systems Curcumin + Docetaxel; Curcumin + Radiotherapy regimens	Standard clinical imaging (CT/MRI)	Clinical trial contexts	Adjunctive therapy for improved outcomes and reduced toxicity (radiation dermatitis reduction)	(222)
5	Breast cancer	Targeted delivery; integrin-mediated uptake; combination therapy	cRGDfK-conjugated PEG-PLGA NPs (with paclitaxel & curcumin)	Near-infrared fluorescence(NIRF) imaging	Preclinical (mouse xenograft)	Targeted fluorescence imaging + enhanced combination therapy	(223)
6	Triple-negative breast cancer	ROS generation; radio-sensitization; pH-responsive drug release	Curcumin–Fe₃O₄@ZIF-8 nanoplatform	T₂-weighted MRI contrast	Preclinical	MRI-guided imaging + ROS-induced apoptosis + radio-sensitization	(39)
7	Cancer (multidrug-resistant cells)	Targeted delivery; intracellular responsive release	Biotin–PEG–poly(curcumin) nanomedicine (with PTX, quantum dots & magnetic NPs)	Fluorescence imaging (QDs) + magnet targeting	Preclinical (cell models)	Dual imaging + drug delivery; overcomes drug resistance	(224)
8	Cancer (4 T1 breast cancer model)	Curcumin delivery to tumor cells; enhanced imaging potential	Fe₃O₄@PCD-Gd coated magnetic NPs with curcumin	T₁ & T₂ MRI contrast	Preclinical (mouse model)	Dual MRI contrast + bioresponsive release + therapeutic efficacy	(225)
9	Breast cancer (magnetic targeting)	MRI + magnetic targeting	Curcumin-loaded magnetic nanoparticles (MNP-CUR)	MRI + magnetic targeting	Preclinical (cell & animal)	Combined anticancer therapy + MRI capability	(107)
10	Oral cancer	Nanocurcumin uptake; fluorescence tracking	Polymeric stabilized nanocurcumin	Autofluorescence imaging (confocal microscopy)	Preclinical (cell)	Fluorescence tracking + anticancer cytotoxicity	(226, 243)

### 5.2 Neurodegenerative disease contexts

**Figure 3:**
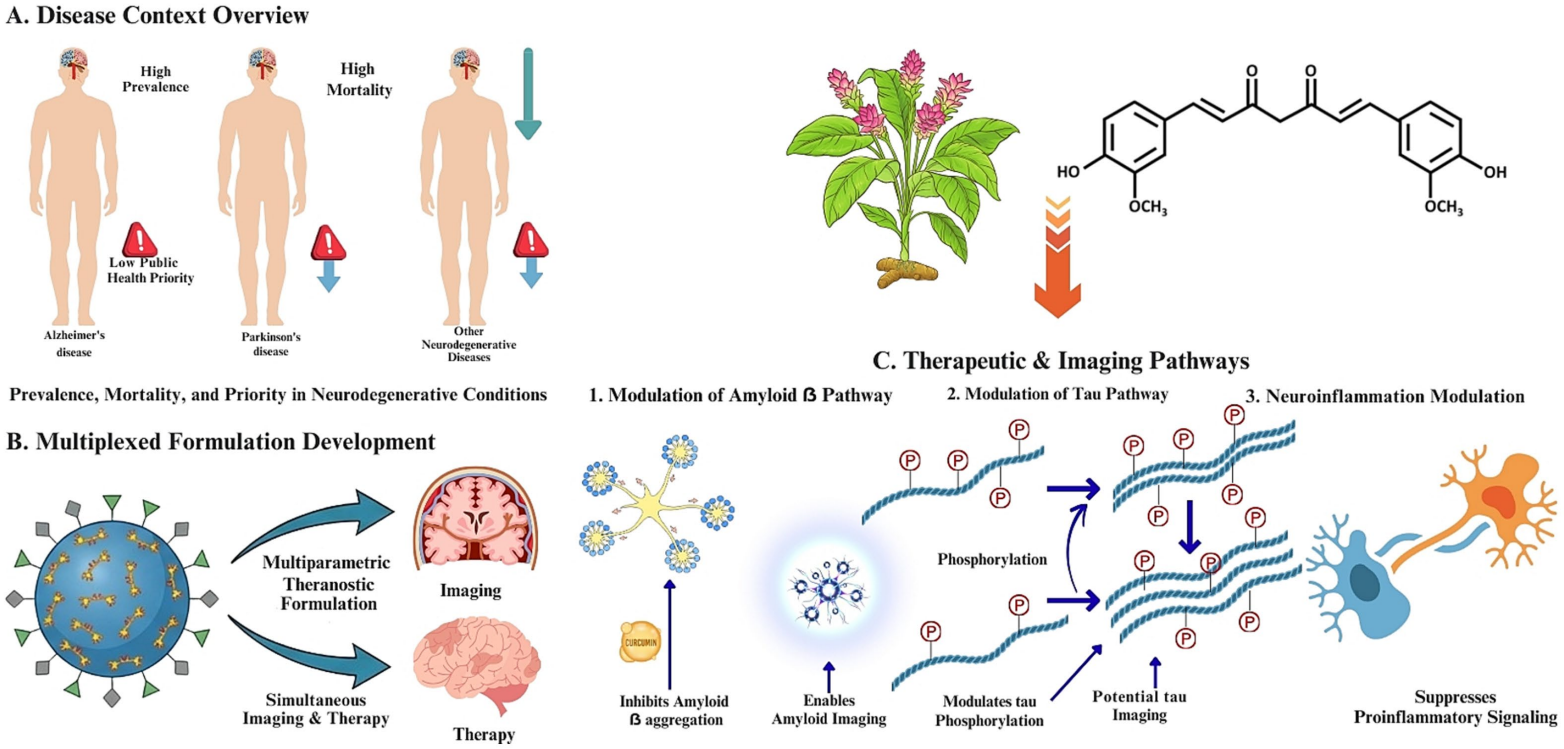
Integrated theranostics framework for neurodegenerative disorders. **(A)** Disease context overview: comparative representation of Alzheimer’s disease, Parkinson’s disease, and other neurodegenerative conditions, highlighting disparities in prevalence, mortality, and public health prioritization. **(B)** Multiplexed formulation development: schematic of a multiparametric theranostics nanoparticle designed for concurrent imaging and therapeutic delivery. **(C)** Therapeutic and imaging pathways: (i) Amyloid β modulation: curcumin-mediated inhibition of amyloid β aggregation and facilitation of amyloid-targeted imaging. (ii) Tau pathway modulation: intervention in tau phosphorylation dynamics with potential for tau-specific imaging. (iii) Neuroinflammation suppression: attenuation of proinflammatory signalling to preserve neuronal function.

Neurodegenerative diseases constitute the third major category of conditions in which curcumin has been investigated as a potential theranostic agent. Neurodegenerative disorders have received comparatively less attention from the curcumin research community (24, 160), a disparity that is highlighted in Figure 3A through disease burden and therapeutic targeting gaps. Nevertheless, mounting evidence demonstrates a suite of valuable imaging and therapeutic effects relevant to these disorders. Curcumin can modulate amyloid β and tau pathways as well as neuroinflammation (Figure 3C); consequently, strategic formulation development on multiplexed curcumin–targeted modulators could yield theranostic agents with high multiparameter imaging correlations and therapeutic coupling (Figure 3B) across multiple neuroinflammatory and neurodegenerative pathways (Table 2) (62, 141).

**Table 2:** Curcumin-based theranostics strategies in neurogenerative disease.

S. no.	Disease area	Target/mechanism	Curcumin analog/platform	Imaging modality/Label	Clinical/preclinical relevance	Theranostic utility	References
1	Alzheimer’s disease	β-Amyloid plaques	[^18^F] Fluorinated Curcumin	PET (^18^F)	Preclinical	Amyloid plaque imaging; early diagnosis and disease monitoring	(227)
2	Alzheimer’s disease	Aβ fibrils (β-sheet binding)	[^125^I]Iodo-Curcumin	BICUR enhanced CT imaging	Preclinical	High-affinity amyloid detection; image-guided intervention	(228)
3	Neurodegeneration	ROS, singlet oxygen, optical sensing	Curcumin & photoactive derivatives	Fluorescence/biosensing	Preclinical–translational	Imaging-guided therapy, ROS sensing, photodynamic theranostics	(80, 229)
4	Alzheimer’s disease	β-Amyloid plaques; neuroinflammation via NLRP3	Curcumin-conjugated nanotheranostic platform	MRI (contrast-enhanced)	Preclinical (AD transgenic models)	Sensitive amyloid detection by MRI; NLRP3 inhibition reverses cognitive deficits	(38)
5	Neurodegenerative diseases (AD)	β-Amyloid aggregation; metal chelation; oxidative stress	Radiolabelled curcumin derivatives	PET / SPECT (^18^F, ^99^ᵐTc, ^68^Ga, ^125^I)	Preclinical	Molecular imaging of amyloid plaques; potential early diagnosis and disease monitoring	(30)

The development of Alzheimer’s disease is primarily determined by the interplay between amyloid-β (Aβ) buildup and tau pathology (239). Aβ is generated through the successive proteolytic cleavage of amyloid precursor protein (APP) by β-secretase (BACE1) and *γ*-secretase, leading to the production of Aβ₄₀ and the more aggregation-prone Aβ₄₂ peptides (73, 161, 162). These peptides oligomerize, form fibrils, and ultimately aggregate as extracellular plaques, resulting in oxidative stress, synaptic dysfunction, and neuroinflammation. Concurrently, tau protein undergoes excessive phosphorylation by kinases such as GSK-3β and CDK5. This induces instability in microtubules and leads to the formation of intracellular neurofibrillary tangles (163–165). Aβ pathology can enhance tau hyperphosphorylation by activating kinases and transmitting inflammatory signals. Tau pathology simultaneously increases neuronal vulnerability, establishing a feedback loop that exacerbates the disease (166, 167).

**Figure 4:**
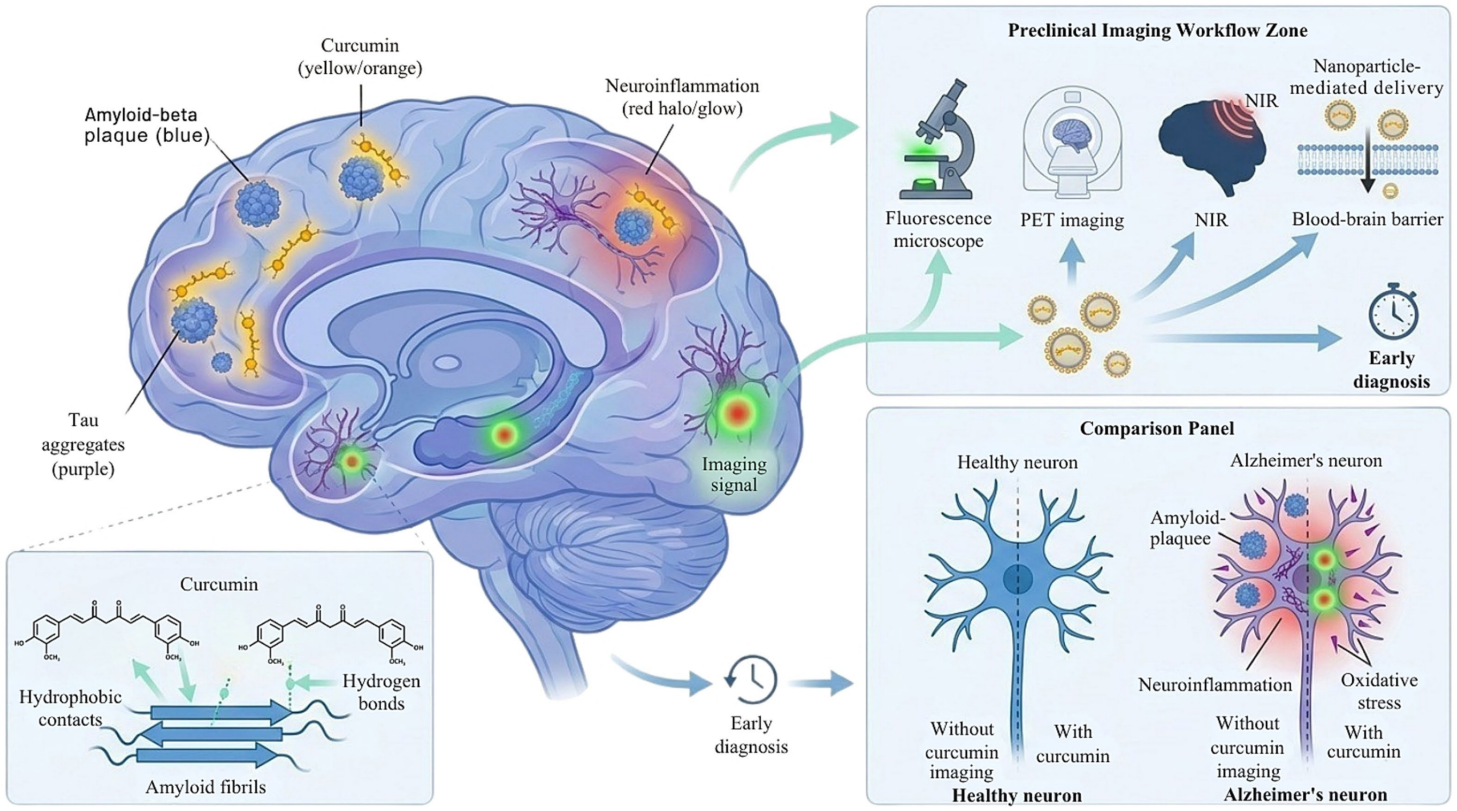
Curcumin-based theranostic imaging in Alzheimer’s disease. Curcumin binds to amyloid-β plaques and tau aggregates through hydrophobic and hydrogen-bond interactions, enabling imaging and modulation of Alzheimer’s disease pathology. The figure illustrates curcumin-mediated fluorescence, PET, and NIR imaging approaches, nanoparticle-assisted blood–brain barrier delivery, and the association of imaging signals with amyloid deposition, neuroinflammation, and oxidative stress in diseased neurons.

Studies have demonstrated that curcumin can alter numerous stages within these pathways. It can inhibit Aβ aggregation, disassemble pre-existing fibrils, reduce β-secretase activity, and diminish oxidative stress and neuroinflammation (141). Curcumin has been demonstrated to diminish tau hyperphosphorylation by modulating kinase activity, namely inhibiting GSK-3β (168). Curcumin addresses both upstream Aβ pathology and downstream tau-mediated neurodegeneration through its multimodal mechanisms, underscoring its potential as a disease-modifying drug (Figure 4) (137, 138).

Curcumin’s classification as a theranostic agent is based on the intrinsic coupling of its imaging and therapeutic functions at the molecular level. Structurally, curcumin exhibits a high affinity for amyloid-β (Aβ) aggregates, enabling its use as a fluorescent probe for plaque detection (169). The imaging signal generated by curcumin reflects its direct binding to pathological aggregates, thereby serving as a real-time indicator of target engagement and therapeutic activity. Notably, this same binding interaction is functionally relevant, as curcumin can inhibit Aβ aggregation and destabilize preformed fibrils, directly modulating the underlying pathology. In parallel, curcumin’s antioxidant and anti-inflammatory properties act on microenvironments associated with Aβ deposition and tau pathology, regions that are also accessible to imaging. This spatial co-localization of signal generation and therapeutic activity provides a mechanistic basis for its theranostic potential. Furthermore, advanced formulations, including radiolabelled derivatives and nanocarrier-based systems, enhance this integration by enabling simultaneous visualization of biodistribution and therapeutic response. Collectively, curcumin operates as a unified theranostic platform in which target engagement, imaging signal generation, and disease modulation occur at the same pathological sites, thereby bridging diagnostic and therapeutic functionalities.

Despite its promise, curcumin’s application in neurodegenerative diseases is limited by poor bioavailability, rapid metabolism, and restricted blood–brain barrier penetration, resulting in low brain exposure. These pharmacokinetic constraints reduce both therapeutic efficacy and imaging reliability. Advanced strategies such as nanoformulations and structural modification are being explored to overcome these limitations. In addition to these pharmacokinetic constraints, several practical challenges arise when curcumin is employed simultaneously as an imaging probe and aggregation inhibitor in complex biological systems.

While curcumin shows promising theranostic potential, its simultaneous use as an imaging probe and aggregation inhibitor presents several challenges in real biological systems. Polydispersity, nanocarrier variability, and differences in curcumin’s aggregation state can affect binding affinity, imaging signal, and inhibitory activity (24, 170). Self-aggregation at higher concentrations may further alter interactions with amyloid-β (Aβ), impacting both imaging accuracy and therapeutic effects (132, 133, 171). Variations in solubility, stability, and release kinetics can lead to inconsistent biodistribution and target engagement. Additionally, limitations in optical properties may reduce *in vivo* imaging reliability (96, 172). Curcumin’s interaction with Aβ, while inhibitory, may also modify plaque structure and complicate quantitative imaging. These factors make it challenging to balance imaging and therapeutic functions. Therefore, optimization of formulation and dosing is essential for reproducible theranostic performance.

### 5.3 Combination strategies with conventional therapies

Combination strategies with conventional therapies remain a central preoccupation for curcumin’s therapeutic use. Where curcumin is not itself administered as the sole intervention, it typically adopts a facilitative role alongside existing regimens (173). As an adjunctive treatment, curcumin can exert potentiating, synergistic, or complementary effects when combined with other pharmaceutical agents (174, 175). Emphasis is placed on both bio-similar and chemically hybrid derivatives of curcumin to formulate poly-drug combinations (176). Sequencing strategies also facilitate combination therapy, enabling the integration of curcumin with established regimens in oncology and neurodegeneration. Such flexibility in engagement with other agents may ultimately contribute to the ongoing personalization of curcumin-based theranostics according to specific disease states and tumour types (177, 178).

Curcumin exhibits a distinct profile compared with established imaging probes and therapeutic agents for neurodegenerative diseases, particularly in terms of specificity, sensitivity, and efficacy. Clinically approved imaging tracers such as Pittsburgh Compound B and Florbetapir F 18 demonstrate high molecular specificity and sensitivity for amyloid-β detection, enabling quantitative and early diagnosis through PET imaging (134–136, 179). In contrast, native curcumin shows moderate specificity due to its multi-target binding nature and comparatively low sensitivity, limited by poor fluorescence properties, rapid metabolism, and suboptimal pharmacokinetics (137, 138). However, structurally modified curcumin derivatives (e.g., CRANAD analogues) have shown improved imaging capabilities in preclinical studies (96). From a therapeutic perspective, conventional agents such as Donepezil provide symptomatic relief, while disease-modifying therapies like Aducanumab target specific pathological hallmarks but with variable clinical efficacy (180). In comparison, curcumin exerts multimodal effects, including inhibition of amyloid aggregation, attenuation of tau hyperphosphorylation, and reduction of oxidative stress and neuroinflammation, although its clinical translation remains constrained by poor bioavailability (160, 181). Collectively, while curcumin lacks the diagnostic precision of established imaging probes and the targeted potency of approved therapeutics, its pleiotropic activity supports its potential as a theranostic scaffold, particularly when optimized through structural modification and advanced delivery systems.

## 6 Preclinical and clinical evidence

The theranostic potential of curcumin has been investigated for both oncological and neurodegenerative diseases through extensive preclinical research, including imaging and therapy evaluations in the same biological systems. Multiple *in vitro* and *in vivo* studies have demonstrated the ability to monitor imaging signals and therapeutic outcomes preclinically, as well as mechanistic correlations (182–185). Several ongoing clinical trials are evaluating curcumin theranostics in humans, though translation has faced challenges related to limited imaging exploration, restricted investigation of bioavailability-enhancing formulations, and a nascent understanding of curcumin modes of action (65, 155). Consequently, monitoring pharmacokinetics, safety, and relevant biomarkers in these trials remains critical for bridging the preclinical–clinical gap and advancing curcumin into human imaging–therapy applications.

Curcumin anticancer properties have been assessed in various *in vitro* and animal models of glioma, breast, prostate, hematopoietic, lung, colorectal, pancreatic, and skin tumours, demonstrating its capability to inhibit proliferation and induce apoptosis, autophagy, and senescence that impede tumour growth (186). Integrated imaging platforms can monitor the delivery of curcumin–bioavailability-enhancing formulations to the brain a critical consideration for sustained release from biodegradable implants while interrogating multiple therapeutic mechanisms (187). Extensive preclinical evidence supports the potential to exploit curcumin as an imaging–therapy agent in tumour intervention.

Curcumin interactions with amyloid-β and tau have been examined in models of Alzheimer’s, Parkinson’s, and Huntington’s diseases, alongside monitoring of neuroinflammation and consideration of blood–brain-barrier permeability, and have established correlations with Aβ, p-Tau, neuroinflammation, and neurotrophic biomarkers in patient biofluids. Imaging platforms enable simultaneous assessment of biodistribution critical given curcumin’s poor lipid–water partition and therapeutic responses, assisting evaluation of emerging bioavailability-enhancing formulations and their combinatorial potential with disease-modifying agents (130, 188). Substantial preclinical support indicates deployment as a theranostic companion for neurodegenerative investigations. Ongoing trials evaluating various curcumin formulations in AD, PD, and MSA reflect the breadth and urgency of exploration (141).

The curcumin theranostic framework has been refined in preclinical investigations targeting both oncological and neurodegenerative diseases. Curcumin–bioavailability-enhancing formulations have been formulated into multiple nanosystems compatible with diverse imaging modalities, and extensive studies have confirmed their ability to facilitate real-time monitoring of biodistribution, pharmacokinetics, and therapeutic responses across diverse cancer, Alzheimer’s, and Parkinson’s preclinical models (173, 189). Investigation of these formulations supports their potential incorporation in human curcumin trials targeting companion-diagnostic and theranostic objectives.

Investigations of curcumin nanoformulations in Alzheimer’s disease indicate that polymeric nanoparticles, lipid-based carriers, and other nano-platforms significantly improve curcumin’s solubility, bioavailability, and ability to penetrate the blood–brain barrier. They also report preliminary clinical findings, including enhanced cognitive scores, decreased biomarkers of oxidative stress and inflammation, and improved tolerance; however, few extensive, long-term trials of specifically defined “nano” curcumin products in Alzheimer’s disease have been conducted (190). Simultaneously, pivotal preclinical research involving a highly stabilized curcumin nanoparticle system evaluated in Tg2576 Alzheimer’s mice demonstrated that the nanoformulation markedly enhanced cue-memory performance in contextual fear conditioning and exhibited more pronounced trends in working-memory assessments compared to standard curcumin or placebo, while *in vitro* findings indicated superior blood–brain barrier permeability (191, 192).

While early-phase clinical trials such as NCT01403545 (liposomal curcumin) and NCT03072992 (intravenous curcumin with chemotherapy) demonstrate safety and therapeutic potential, only limited studies (e.g., NCT01383161) have incorporated imaging endpoints, and none have evaluated fully integrated curcumin-based theranostic platforms. Representative studies highlighting this translational disparity are summarized in Table 3. The preclinical and early clinical findings indicate that nanoformulated or highly bioavailable curcumin may possess significant translational potential in Alzheimer’s disease; however, the clinical evidence remains constrained by small sample sizes, heterogeneous formulations, and inconsistent dosing regimens. Consequently, larger, rigorously controlled trials specifically designed around defined nano-curcumin platforms are essential.

**Table 3:** Representative preclinical and clinical studies supporting curcumin-based theranostics.

Study (Ref./Trial ID)	Model	Formulation	Key outcome	Translational relevance
Kakkar et al. (20)	Rat (brain delivery)	Curcumin-loaded solid lipid nanoparticles (C-SLNs)	~30-fold ↑ brain AUC; improved bioavailability	Demonstrates feasibility of CNS-targeted nanocarriers
Jain et al. (230)	Tumor-bearing animal models	Magnetic nanoparticle–curcumin conjugates	MRI contrast + anticancer activity	Proof-of-concept for theranostic (therapy + imaging) systems
NCT01403545	Healthy volunteers (Phase I)	Intravenous liposomal curcumin	Demonstrated safety, tolerability, and pharmacokinetics	First clinical evidence for nanoformulation feasibility (ClinicalTrials.gov)
NCT03072992	Advanced/metastatic breast cancer (Phase II)	Intravenous curcumin (CUC-01) + paclitaxel	Evaluated ORR, PFS, safety; combination therapy approach	Shows clinical use of IV curcumin, but no imaging integration (ClinicalTrials.gov)
NCT01383161	Mild cognitive impairment (Phase II)	Oral bioavailable curcumin (Theracurmin^®^)	Included PET imaging (FDDNP) and MRI; cognitive assessment	Rare example integrating imaging endpoints in humans (ClinicalTrials.gov)
NCT03980509	Breast cancer patients (Phase I)	Oral curcumin extract	Assessed tumor biomarkers (Ki-67, apoptosis)	Demonstrates biological activity but lacks imaging/targeted delivery (ClinicalTrials.gov)
NCT03865992	Breast cancer survivors (Phase I)	Oral curcumin	Evaluates symptom relief (joint pain)	Illustrates non-oncologic, supportive use; not theranostic (ClinicalTrials.gov)
NCT03769766	Prostate cancer (active surveillance)	Oral curcumin vs. placebo	Evaluates disease progression	Highlights preventive/adjunct role, not imaging-based (ClinicalTrials.gov)

### 6.1 *In vitro* and *in vivo* models

*In vitro* and *in vivo* models have been extensively used to assess curcumin’s theranostic potential in the context of cancer and neurodegenerative disorders. For cancer applications, curcumin-mediated alterations in biological pathways and preclinical response have been evaluated in various cell lines and animal models, as summarized elsewhere (155, 193). The compounds targeting specific biological processes, such as NF-κB and COX-2, can serve as surrogate imaging endpoints. Corresponding *in vitro* tests permit the identification of candidates that can suitably probe pharmacodynamic changes and determine optimal dosing regimens, thereby enhancing therapeutic efficacy (194, 195). Preclinical studies have employed glioblastoma-bearing rat models and multimodal imaging modalities to investigate the anticancer effects of curcumin and to assess the impact of dietary curcumin on radio-sensitization. Combinations of curcumin with irradiation and other modalities have been used to investigate potential synergies; these synergies are more challenging to achieve when the drugs are administered at different times (196, 197).

Preclinical studies addressing neurodegenerative contexts have characterized formulations and mechanisms in cellular systems, such as by measuring β-aggregate load in Aβ-expressing cells and tracking myelination changes after curcumin treatment (198). Established animal models have also been exploited to demonstrate therapeutic effects. Validation of formulation choices, imaging signal enhancement, and treatment effects has been accomplished using screening methods; depending on the context, biodistribution can require further investigation (198–200). A well-controlled clinical trial of curcumin coupled with pharmacokinetic characterization has been reported; the specified protocol and analysis drawings for regimen optimization warrant broader consideration (201, 202). Regulatory challenges persist for the introduction of theranostic pairs. The therapeutic application of curcumin remains within exploratory stages, and attention has been directed to screening, formulation design, and mechanism elucidation (13).

### 6.2 Clinical trials and translational challenges

Despite extensive preclinical research supporting curcumin’s theranostic application and the urgent need for novel imaging–therapeutic solutions in cancer and neurodegeneration, clinical progression has been limited (176). Existing human studies focus on unmodified curcumin as a dietary supplement or alternative medicine in advanced-stage brain or breast cancer or in combination with chemotherapy, radiation, or biological agents for glioma, breast cancer, or glioblastoma (203). Imaging endpoints such as fluorescence, magnetic resonance, or PET have not been assessed. Translational barriers stem from the considerable discrepancy between preclinical formulations and clinical practice (204, 205). Difficulty in obtaining regulatory approval for complex formulations has impeded clinical exploration of curcumin nanoparticles or liposomes. Fully subsidizing or recovering formulation costs becomes impractical for many researcher-initiated initiatives, highlighted by the lack of a consolidated platform that enables universal project adaptation (206).

The discrepancy between preclinical formulations and clinical practice in curcumin-based theranostics arises from critical differences in formulation design, dosing strategies, delivery systems, and imaging integration. In preclinical studies, curcumin is commonly administered using optimized nanocarriers such as liposomes, polymeric nanoparticles, or surface-functionalized systems engineered for enhanced stability, target specificity, and imaging capability (50–52). These systems often employ controlled dosing regimens and specialized routes of administration (e.g., intravenous or intranasal) to maximize bioavailability and tissue accumulation. In contrast, clinical studies have predominantly utilized unmodified curcumin or simple oral formulations, which exhibit poor solubility, rapid metabolism, and limited systemic exposure (12, 24, 44). This pharmacokinetic limitation, coupled with the absence of radiolabelled or contrast-enhanced formulations, explains why imaging endpoints such as fluorescence, MRI, or PET have not yet been incorporated into human studies. Furthermore, advanced nanocarrier systems face challenges in scale-up and standardization under Good Manufacturing Practice (GMP) conditions, restricting their clinical adoption. Consequently, these differences lead to reduced therapeutic efficacy and lack of measurable imaging signals in humans. Bridging this gap requires the development of clinically scalable, regulatory-compliant nanocarriers and imaging-enabled formulations that retain the functional advantages demonstrated in preclinical systems.

#### 6.2.1 Regulatory and economic challenges in nanotheranostic translation

Regulatory and economic barriers remain major impediments to the clinical translation of complex formulations such as nanoparticles and liposomes in curcumin-based theranostics (13). A primary challenge lies in their classification as combination products, necessitating simultaneous evaluation of pharmacological efficacy and imaging performance, often across multiple regulatory frameworks (13, 207, 240). In addition, the absence of harmonized guidelines for nanomedicine, particularly regarding long-term toxicity, biodistribution, and immunogenicity, complicates approval pathways. Concerns related to batch-to-batch reproducibility and scale-up further limit industrial adoption, given the sensitivity of nanosystems to minor process variations. From an economic perspective, high manufacturing costs, reliance on specialized equipment, and the need for dual therapeutic–diagnostic validation substantially increase development expenditure. Moreover, limited reimbursement frameworks and uncertain commercial returns reduce industry incentives. Addressing these barriers requires early regulatory engagement, implementation of Quality by Design approaches, and adoption of scalable manufacturing techniques such as microfluidics. The use of biocompatible, regulatory-accepted materials and clinically validated imaging biomarkers may further streamline development. Collectively, aligning formulation design with regulatory expectations and economic feasibility is essential to bridge the gap between preclinical promise and clinical application.

Curcumin possesses numerous inherent constraints that constrain its medicinal and imaging applications, although it’s promising biological activity. The primary constraint is exceedingly low oral bioavailability, attributed to inadequate aqueous solubility in the gastrointestinal tract, restricted permeability through the intestinal epithelium, significant first-pass metabolism (notably glucuronidation and sulfation), and swift systemic elimination, collectively leading to less than 1–2% of ingested curcumin entering systemic circulation in its active form (58, 176). Curcumin is chemically unstable at physiological pH, rapidly disintegrating in neutral or alkaline environments, and its active metabolites (such as tetrahydrocurcumin and hexahydrocurcumin) frequently demonstrate diminished or modified pharmacological activity relative to the parent substance (176, 208, 209). Additional inherent limitations encompass non-specific, pleiotropic pharmacology (binding to multiple targets with moderate affinity), complicating dose–response relationships and mechanistic interpretation, as well as a propensity to chelate metal ions like iron, which raises concerns regarding potential interference with iron absorption and hematological effects at elevated or chronic doses (208, 210, 211). Encapsulation into sophisticated nanocarriers (e.g., PLGA nanoparticles, nanostructured lipid carriers, liposomes, mesoporous silica, and biomimetic exosomes) significantly improves curcumin’s aqueous solubility, protects it from rapid degradation and metabolism, and prolongs systemic circulation. These alterations collectively enhance bioavailability to clinically significant levels while promoting passive tumor accumulation through the EPR effect. “Intelligent” designs featuring stimuli-responsive components (pH-, redox-, or enzyme-activated release) and active targeting ligands (e.g., folate, transferrin, hyaluronic acid, RGD peptides) facilitate spatiotemporal regulation of drug release, markedly enhancing tumor-specific absorption and reducing off-target toxicity (31).

## 7 Safety, toxicity, and regulatory considerations

Theranostics relates to the development of imaging and therapeutic agents that aim to improve cancer management. Cancer continues to be one of the leading causes of death worldwide despite the significant progress made in its diagnosis and treatment (206, 212, 213, 238). Cancer is characterized by uncontrolled cell division and the ability to invade surrounding tissues. Curcumin, a polyphenolic compound derived from *Curcuma longa*, has shown enormous potential in the treatment of cancer as well as several other diseases such as Alzheimer’s (214, 215). The World Health Organization (WHO) emphasized on the need for identifying safe, cheap and efficacious compounds for treatment and control of brain tumours. Curcumin fits these requirements (216). The compound exhibits an adequate safety profile, acts as an immunoadjuvant and is involved in protecting from oxidative stress. It can be safely consumed by human beings and is considered to be non-toxic (216, 217). A study conducted to find out the potential of curcumin to cross the blood–brain barrier establishes that curcumin can penetrate at nearly 0.2% of its total dose (217). Curcumin’s ability to cross the blood–brain barrier makes it suitable for treating brain tumours and central nervous diseases. It is now considered that there is an urgent need for curcumin-based cross-modality theranostic agents that can actively target certain cancers and Central Nervous System (CNS) diseases (218, 219).

## 8 Knowledge gaps and research outlook

As theranostic research involving curcumin advances, several essential knowledge gaps and future directions remain. The significant cohort of preclinical studies demonstrating the potential benefits of curcumin as both an imaging agent and an anticancer or anti-Alzheimer therapeutic has not been met with an equally robust number of clinically relevant experiments addressing theranostics in either disease (218). Within the curcumin theranostic literature reviewed here, specific opportunities for further exploration include: greater elucidation of the impact of formulation on therapeutic activity when employing curcumin-based biologically active imaging agents; a more comprehensive investigation of the multimodal curcumin theranostic proposal linking optical imaging with electrochemical modification of curcumin to extend beyond cell cultures and single anatomical locations; and systematic exploration of combinatorial anticancer regimens including the imaging moiety of formulated curcumin, which may unlock additional theranostic potential and impact the design of imaging-based clinical studies (216). Given curcumin’s extensive use as a food and dietary ingredient, the establishment of administered dose thresholds in the context of imaging–therapeutic pairs that can be directly compared with existing formulations would further strengthen preliminary materials supporting theranostics. Confluence of these many paths toward refining curcumin as an imaging-guided therapeutic warrants timely consideration, not only in view of their parallel relevance to curcumin but also to foster synergy across complementary interests encompassing theranostic agents, procedures, and formulation techniques focused on diverse substances and traits (69, 218, 219).

## 9 Conclusion

Curcumin is a promising natural scaffold for theranostics, integrating diagnostic imaging and therapeutic action within a single platform. Its intrinsic fluorescence and affinity for β-sheet-rich amyloid structures enable target-specific imaging, while the same interactions contribute to therapeutic effects through inhibition of aggregation, modulation of tau phosphorylation, and reduction of oxidative stress and neuroinflammation. This mechanistic coupling distinguishes curcumin from conventional agents that function solely as either probes or therapeutics.

However, clinical translation remains limited by poor bioavailability, rapid metabolism, low tissue accumulation, and suboptimal imaging sensitivity. A significant gap persists between advanced preclinical formulations—such as nanoparticle-based and radiolabelled systems—and the simpler forms evaluated in clinical studies. Regulatory complexity, scalability challenges, and the lack of validated imaging endpoints further constrain progress.

Recent advances in nanocarriers and multimodal imaging systems offer viable strategies to overcome these barriers by improving pharmacokinetics, target specificity, and real-time monitoring of therapeutic response. Future progress will depend on developing clinically scalable formulations, integrating quantitative imaging endpoints into trials, and strengthening mechanistic correlations between imaging signals and therapeutic outcomes. Overall, curcumin remains a strong candidate for next-generation theranostic development.

Collectively, the evidence indicates that curcumin’s value lies not merely in its individual diagnostic or therapeutic properties, but in its unique ability to unify these functions within a single, mechanistically linked platform.
